# Corn Silk Tea for Hypertension: A Systematic Review and Meta-Analysis of Randomized Controlled Trials

**DOI:** 10.1155/2019/2915498

**Published:** 2019-01-17

**Authors:** Shihua Shi, Siming Li, Weihao Li, Hao Xu

**Affiliations:** ^1^Hospital of Chengdu University of Traditional Chinese Medicine, Chengdu, Sichuan Province 610072, China; ^2^Cardiovascular Diseases Center, Xiyuan Hospital, China Academy of Chinese Medical Sciences, Beijing 100091, China; ^3^Cardiology Division, West China Hospital, Sichuan University, Chengdu, Sichuan Province 610041, China

## Abstract

Corn silk, a traditional Chinese medicine, has been found to exert an antihypertensive effect in clinical practice and trials. However, systematic review of current evidence on this topic was not available. Thus, this study aims to assess safety and efficacy of corn silk tea (CST) in improving clinical outcomes in patients with hypertension. A systematic literature search was conducted through popular electronic databases up to October 2018. Randomized controlled trials (RCTs) comparing CST plus conventional antihypertensive drugs with conventional antihypertensive drugs alone were included. The main outcome was total blood pressure lowering efficacy. The risk of bias assessment according to the Cochrane Handbook was used to evaluate the methodological quality of the included trials. Review Manager 5.3 software was used for data analyses. Five RCTs involving 567 participants were included. Due to the poor quality of methodologies of most trials, limited evidence showed that CST plus antihypertensive drugs might be more effective in lowering blood pressure compared with antihypertensive drugs alone (RR = 1.27; 95% CI: 1.17 to 1.38,* P*<0.00001; heterogeneity:* P *= 0.51,* I*^*2*^ = 0%, fixed‐effect model). However, there is no evidence that CST plus conventional antihypertensive drugs has less adverse events than conventional antihypertensive drugs.

## 1. Introduction

High blood pressure (BP), as one of the most common diseases in the world [1], has been recognized as a major risk factor for cardiovascular and cerebrovascular diseases, leading to major health complications such as heart attack, strokes, and renal dysfunction [2–6]. One quarter of the world's adult population has hypertension [7] and the high prevalence of hypertension makes it a significant factor for mortality and morbidity [8, 9]. Controlling hypertension is of paramount importance for public health [10–12]. Today, phytotherapy is commonly used to treat hypertension in China and the West [13–15]. Chinese and Native Americans used corn silk tea (CST) to lower BP for centuries, but there is a lack of reliable evidence of efficacy regarding CST treatment for hypertension all along.

Corn silk (*Maydis stigma*) is a waste material from corn cultivation, but it is also an inexpensive medical diet of plant [16]. Corn silk (CS), as a traditional Chinese classical herb, was first recorded in the medical classic* Materia Medica of South Yunnan* by the Chinese physician Lan Mao (1397–1470) during the Ming dynasty of China. CS is considered an important medicinal plant, with the function of inducing diuresis and excreting dampness, alleviating syndrome of internal stagnation of fluid-dampness according to traditional Chinese medicine (TCM) theory [17]. CST has been claimed to have many benefits to human health such as decreasing inflammation, reducing edema, improving obesity, and lowering BP [16–20]. Recently, there has been a focus on the role that CS can play in the treatment of hypertension. Several studies on CST and its health benefits on hypertension have been published [21–25] and the current evidence on its safety and efficacy has not been summarized. In this study, we conducted a systematic review and meta-analysis of randomized clinical trials (RCTs) to clarify the clinical efficacy of CST on BP.

## 2. Methods

### 2.1. Search Strategy

This systematic review is conducted in accordance with the Preferred Reporting Items for Systematic Reviews and Meta Analyses: the PRISMA Statement [26]. Relevant publications were electronically searched in 7 databases from the start date of the databases until October 31, 2018, including PubMed, Embase, Science Direct, Springer Link, China National Knowledge Infrastructure Database (CNKI), Chongqing VIP Chinese Science and Technology Periodical Database (VIP), and Wanfang database. There was no restriction on publication status and publication language. The following search terms were used: “hypertension” OR “high blood pressure” for hypertension, and “corn silk” OR “Maydis stigma” OR “Zea mays hairs” for corn silk, and “clinical trial” OR “randomized controlled trial” for RCTs.

### 2.2. Selection Criteria

This review included RCTs that met the following criteria: (1) only patients with hypertension were included. Hypertension should be diagnosed based on the criteria documented in the seventh report of the Joint National Committee or other well-accepted guidelines and definitions [27]. (2) RCTs that examined the effect of CST in combination with pharmaceutical treatment comparing with pharmaceutical treatment were identified. Participants in the treatment group should be treated by CST combined with pharmaceutical treatment. Participants in the control group were treated by pharmaceutical treatment alone. The pharmaceutical treatment used in the CST group should be the same as the controls. The CST treatment includes CST or CS decoction. (3) The studies must have assessed the total antihypertensive effective rate as a result and reported the total effective cases. There were no restrictions in terms of gender, ethnicity, blinding, or treatment duration.

The exclusion criteria were as follows: (1) other therapies that were used in either the CST group or control group; (2) if the efficacy of CST on hypertension was not reported; (3) animal studies; (4) duplicate publications.

### 2.3. Study Selection and Data Extraction

All titles, abstracts, and full‐text articles were reviewed by two authors (Shihua Shi and Siming Li) independently, based on the eligibility criteria listed above. Data were extracted by the two authors on their own, and the extracted details include the following information: the name of the author, publication time, the age of the patients, sample size, diagnosis criteria of hypertension, baseline difference and study design involving methodologic quality, interventions in the CST and control groups, compositions and dosage of CST, duration of therapy, and adverse events. Disagreements between the two authors were resolved by discussion and if needed, arbitrated by another viewer (Hao Xu).

### 2.4. Quality Assessment and Data Synthesis

The methodologic quality of the eligible trials was assessed according to the Cochrane Collaboration's tool [28]. Comparison between CST and antihypertensive drugs and antihypertensive drugs alone was performed in this study. Outcome measures after treatment were presented as risk ratio (RR) with 95% CI for dichotomous outcomes. Heterogeneity of effect sizes was measured using the* I*^*2*^ statistics. If substantial heterogeneity was observed, we used random-effects model to assess the effects of CST for hypertension across trials (*I*^*2*^ > 50% or* P* < 0.1) or else a fixed-effects model was adopted. All of data in this meta-analysis were conducted in the Review Manager software (RevMan, Version 5.3, Copenhagen: The Nordic Cochrane Centre, The Cochrane Collaboration, 2014).

## 3. Results

### 3.1. Study Selection

**Figure 1:**
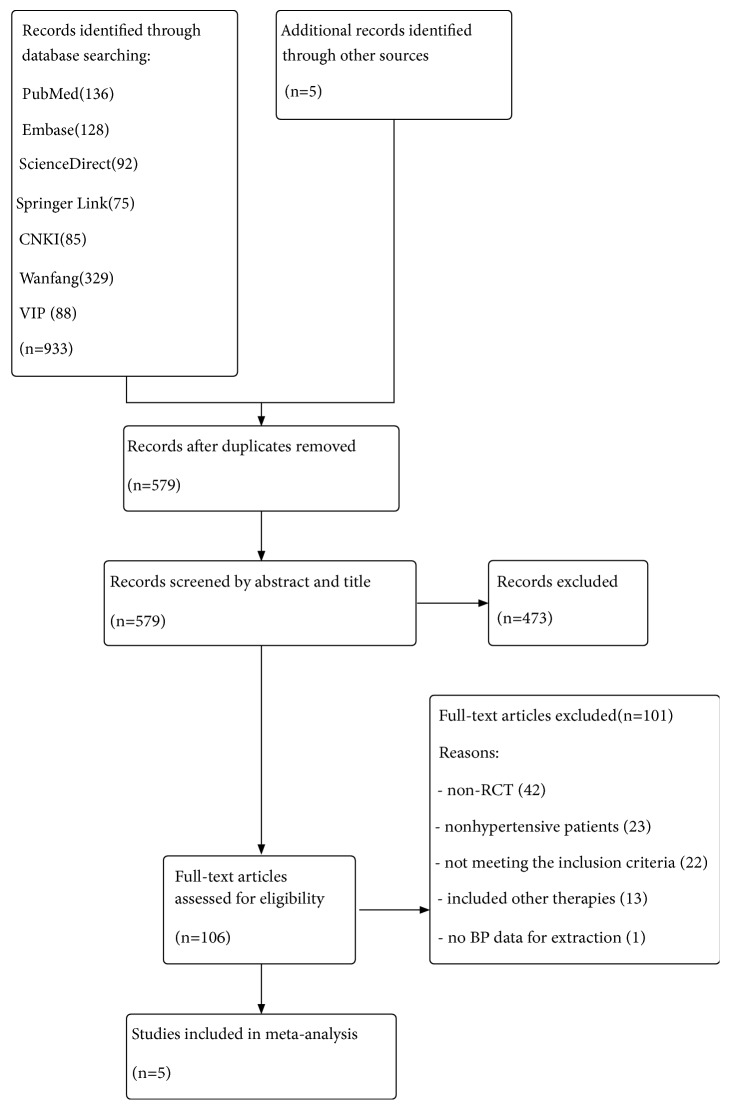


Among the 938 studies identified in the initial search, 359 duplicate publications were excluded. After reading the titles and abstracts, 106 full-text articles were assessed eligible. 65 articles were excluded because they were non-RCT or nonhypertensive patients. Then, we excluded 36 trials because of the following reasons: 22 articles did not meet the inclusion criteria; intervention in 13 articles included other herbal therapies; and 1 article had no BP data for extraction. Ultimately, 5 eligible studies including a total of 567 patients with hypertension were analyzed [21–25]. A summary of the study selection is presented in the PRISMA flow chart (Figure 1).

### 3.2. Study Characteristics

The descriptive information of the five included studies was showed in Table 1. All 5 RCTs were single-center studies conducted in China and published in Chinese between 2009 and 2017. The sample size ranged from 64 to 206 with a mean size of 113. All patients enrolled were Chinese. All patients included were diagnosed with hypertension, which was based on criteria of World Health Organization-International Society of Hypertension Guidelines for the Management of Hypertension-1999 [22], Chinese Guidelines for the Management of Hypertension-2010 (CGMH-2010) [23], Chinese Guidelines for the Management of Hypertension-2005(CGMH-2005) [24], and Chinese Guidelines for the Management of Gestational Hypertension-2012 (CGMGH-2012) [21, 25]. The age of the enrolled patients ranged from 20 to 84 years old. No significant difference on baseline was identified in all the studies. The duration of treatment ranged from 1 week to 12 weeks. No study reported the dropouts and source of funding. Interventions of CST and antihypertensive drugs were all given orally. The components of CST in each trial were summarized in Table 2. The serum homocysteine (HCY) level [23] and edema [21] were reported in 1 trial. The outcome of urine protein was reported in 2 trials [21, 25]. Adverse events were not reported in 5 trials [21–25].

### 3.3. Methodologic Quality

**Figure 2:**
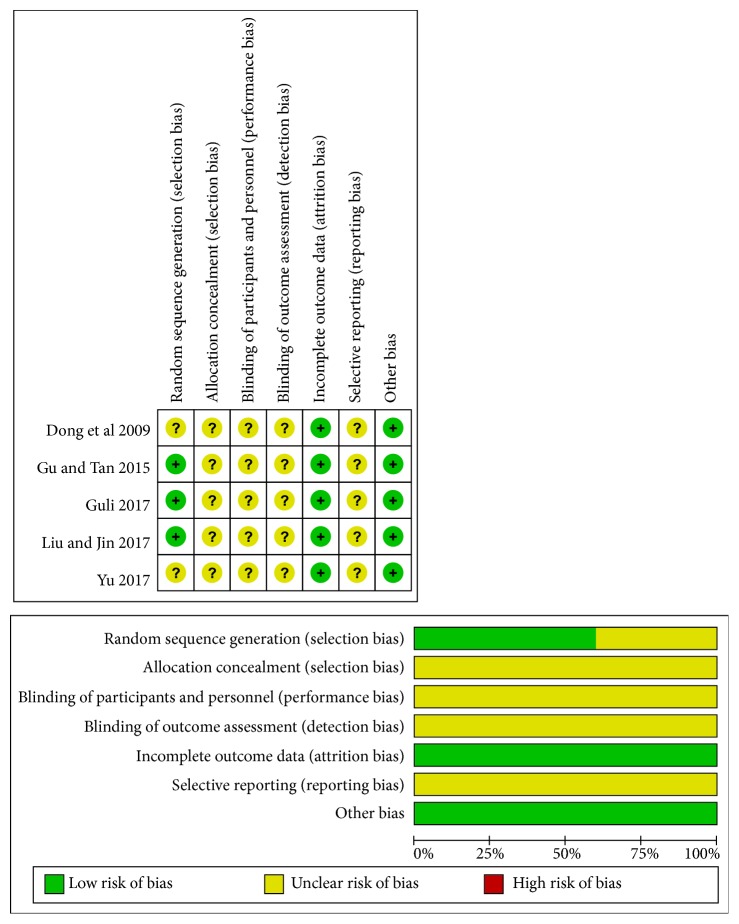


The assessment of methodologic quality of each involved study was shown in Figure 2. Three trials declared the generation of the random sequence among them [21, 23, 25], whereas the other 2 trials only mentioned randomization without detailed information in the text [22, 24]. Details concerning concealment of allocation and blinding of patients, investigators, and assessors were unclear in all the trials. No study reported dropouts and long-term follow-up. Furthermore, selective reporting cannot be evaluated.

### 3.4. Outcome Measures

**Figure 3:**
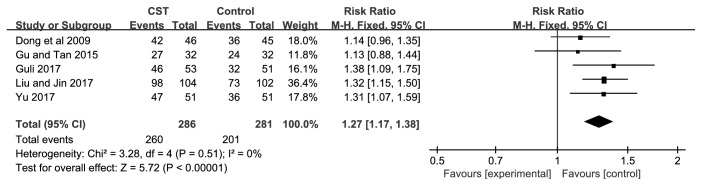


The effectiveness of CST on BP was evaluated in all of the 5 trials [21–25]. There were 286 patients in the CST groups and 281 patients in the control groups, respectively. A fixed-effects model was used for statistical analysis based on the test of heterogeneity (BP: chi-square = 3.28,* P *= 0.51,* I*^*2*^ = 0%). The combined effects of these 5 independent trials showed a significant lowering effects of CST plus antihypertensive drugs on BP in patients when compared with antihypertensive drugs alone (RR=1.27; 95% CI:1.17 to 1.38,* P*<0.00001) (Figure 3).

We found one study evaluated the effectiveness of CST plus antihypertensive drugs on edema [21] and HCY [23]. The outcome of urine protein was reported in two studies [21, 25]. There were 104 patients in the CST group and 102 patients in the pharmaceutical group when researching the efficacy of CST on edema. 32 participants were included in the CST group and the control group respectively when studying the curative effect on HCY. In the treatment group and control group, 157 patients and 153 patients were included respectively when studying urine protein. A noteworthy reduction of edema, urine protein, and HCY in favor of CST therapy was observed after treatment in terms of the few clinical trials.

The outcome of serious adverse events was not mentioned in 5 trials [21–25]. Nothing was reported about severe adverse effects in patients treated by either CST plus conventional antihypertensive drugs or conventional antihypertensive drugs alone.

## 4. Discussion

We originally intended to study the efficacy of CST on hypertension with all-cause mortality and cardiovascular death as the primary outcomes in fact. The secondary outcomes were antihypertensive effect and adverse events. However, several studies on CST and its health benefits on hypertension to date have not reported all-cause mortality and cardiovascular death. These trials mainly studied the total blood pressure lowering efficacy of CST plus conventional antihypertensive drugs comparing with conventional antihypertensive drugs alone. The current evidence on its safety and efficacy has not been summarized. Considering all above, we ultimately conducted this systematic review and meta-analysis of RCTs to clarify the clinical efficacy of CST plus conventional antihypertensive drugs on BP, where the main outcome was total blood pressure lowering efficacy.

A total of five RCTs involving 567 hypertensive patients without specific ethnic characteristics met the inclusion criteria in this review. In general, the pooled analyses of the current RCTs might suggest that the combination of CST and conventional antihypertensive medicine treatment may have a better effect on total antihypertensive effective rate than conventional pharmaceutical treatment alone in patients with hypertension (RR = 1.72; 95% CI:1.45 to 2.04,* P*<0.00001; heterogeneity:* P* = 0.43,* I*^*2*^ = 0%, fixed‐effect model).

This finding may mean that using CST as an adjuvant phytotherapy in treating hypertension is likely to have higher hypotensive effective rate. In addition, doctors could probably use this result to give patients advice on phytotherapy during treatment of hypertension.

Unfortunately, our review could not provide valid evidence that CST improves edema, urine protein, and HCY, though a few trials revealed the beneficial role of CST combined with antihypertensive drugs on them clinically. These few studies were not strong enough to give the answer whether CST exerts positive effect on edema, urine protein, and HCY statistically. More relevant studies with better quality would be required for the statistical significance and further review.

Given literature searches found no review focused on the effect of CST for hypertension, this systematic review and meta-analysis researched on the clinical efficacy of CST for hypertension for the first time, suggesting that CST plus antihypertensive drugs appeared to be more effective in lowering BP compared with antihypertensive drugs alone. Besides, comparing with other Chinese medicine decoctions, CST has the advantages of good taste, low price, and good availability. For these reasons above, treating hypertension with CST as an adjuvant phytotherapy is easy to implement and the patients with hypertension are likely to have better compliance. Moreover, no solid laboratory evidence of how CST works in hypertension has been published, though some progress has been made in demonstrating the mechanisms of BP lowering effect of TCM [29–32], which may suggest an interesting direction for further study.

Before accepting the positive findings above, some limitations should be considered. Firstly, databases published in other languages except Chinese and English were not included in our study. Therefore, a certain degree of potential selective bias might exist and may limit the generalization of the evidence. Since all studies were conducted in China, we were not able to demonstrate whether this result can be reproduced in other parts of the world partly due to undemonstrated effect mechanisms and difference in ethnic, dietary, and social-economic characteristics. Secondly, positive results are easier to be published [33] and the efficacy of CST for hypertension might be overestimated. Thirdly, the methodologic quality is poor according to the Cochrane Collaboration's tool, which is the inherent shortcoming in our primary studies. For example, only three trials [21, 23, 25] provided adequate sequence generation methods, although all studies declared that participants were randomized into the CST group and control group. Besides, no trials reported the concealment of allocation and no studies compared CST with placebo. Unfortunately, no adverse events were reported in all trials and the safety of CST was rarely reported. It may be because no adverse events occurred actually or the researchers believed CS is nonpoisonous and fairly safe based on the theory of “drug homologous food” in TCM and there is no need to pay attention to the serious adverse events of CS. In view of this, the safety of CS used in clinical treatment still needs to be further studied. Given the poor methodological quality of the evidence and inadequate reporting currently available, the credibility of the clinical evidence of CST in the present study might be undermined.

## 5. Conclusion

Though people have applied CST or CS decoction for many treatments for decades in China, Korea, Vietnam, America, and some other countries [34, 35], to our knowledge, there has not been any systematic review and meta-analysis to value the clinical effects of CST on hypertension, offering the summarized evidence of efficacy. The present study is the first of its kind to provide an evidence-based approach to the CST treatment of hypertension, which should be given priority for future preclinical and clinical studies. In summary, CST plus antihypertensive drugs could be more effective on lowering blood pressure than conventional antihypertensive drugs alone, accordingly suggesting CST may be a new alternative natural-based treatment for hypertension, although some limitations might weaken the validity of positive findings considering the poor methodological design. From a clinical point of view, well-designed phytotherapy trials with high methodological quality are needed to validate the effect of CST for patients with hypertension.

## Figures and Tables

**Table 1:** Basic characteristics of the included trials.

**References**	**Sample size (T/C)**	**Age (years)**	**Diagnostic criteria**	**Intervention**	**Control**	**Treatment duration**
Dong et al. 2009 [24]	46/45	49.7±14.5	CGMH-2005	CS 60g/d + C	Hypotensive drugs	12 weeks
Yu 2017 [22]	51/51	57.2±3.5	CGMH-2005	CS 60g/d + C	Nifedipine controlled released tablets (0.03g q.d.)	12 weeks
Gu and Tan 2015 [23]	32/32	65.1± 8.8	CGMH-2010	Modified CST (CS 10g)+ C	Enalapril tablets (10mg q.d.) Folic acid tablets (0.8mg q.d.)	8 weeks
Liu and Jin 2017 [21]	104/102	30±0.5	CGMGH-2012	CS 50g/d + C	Hypotensive drugs	1 weeks
Guli et al. 2017 [25]	53/51	29±0	CGMGH-2012	CS 50g/d + C	Hypotensive drugs	1 weeks

CN: China; CS: corn silk; CST: corn silk tea; T: treating group; C: control group; CGMGH: Chinese Guidelines for the Management of Gestational Hypertension; CGMH: Chinese Guidelines for the Management of Hypertension.

**Table 2:** Components of CST used in the included trials.

**References**	**CST**	**Components and directions**
Dong et al. 2009 [24]	CST	corn silk 60g/d, tid
Yu 2017 [22]	CST	corn silk 30g/dose, 2 dose/day
Gu and Tan 2015 [23]	modified CST	corn silk10g, Ganoderma lucidum 20g/dose, 1 dose/day
Liu and Jin 2017 [21]	CST	corn silk 30~50g, 1 dose/day
Guli et al. 2017 [25]	CST	corn silk 30~50g, 1 dose/day

CST = corn silk tea.
